# Ion-specific effects on the sonochemical degradation of Naphthol Blue Black: from inorganic salts to natural water matrices

**DOI:** 10.1016/j.ultsonch.2026.108105

**Published:** 2026-10-09

**Authors:** Hamza Ferkous, Muthupandian Ashokkumar, Oualid Hamdaoui

**Affiliations:** a Department of Process Engineering, Faculty of Technology, Badji Mokhtar-Annaba University, 23000 Annaba, Algeria; b School of Chemistry, The University of Melbourne, Parkville, Victoria 3010, Australia; c Chemical Engineering Department, College of Engineering, King Saud University, 12372 Riyadh, Saudi Arabia

**Keywords:** Naphthol blue black, Sonochemical degradation, High-frequency ultrasonication, Inorganic salts, Natural water matrices, Cavitation

## Abstract

This study investigates the effects of inorganic salts and natural water matrices on the high-frequency sonochemical degradation of Naphthol Blue Black (NBB). Experiments were conducted at 585 kHz and 3.58 W/cm^2^ using initial NBB concentrations of 5 mg/L (8.11 × 10^−6^ M) and 30 mg/L (4.87 × 10^−5^ M). NaCl, KNO_3_, Na_2_SO_4_, and NaHCO_3_ were evaluated over broad concentration ranges and compared with bicarbonate-rich Ca–Mg mineral water and seawater. The response depended strongly on ion identity and pollutant loading. Chloride produced the most persistent enhancement, nitrate showed a more modest positive trend, sulfate displayed an optimum-type response, and bicarbonate shifted from promotion at low concentration and low NBB loading to pronounced inhibition at higher concentrations. Both natural matrices enhanced NBB removal relative to ultrapure water, with seawater showing the greatest improvement. The observed trends are consistent with combined contributions from pollutant accessibility to the bubble–liquid interface, ion-dependent radical chemistry, and changes in the cavitation environment. These findings demonstrate the importance of realistic water chemistry when evaluating high-frequency sonochemical treatment of saline and mineralized waters.

## 1 Introduction

Synthetic dyes remain a major class of aquatic pollutants because they are intentionally designed to resist fading and chemical breakdown, and substantial fractions of applied dyes can be discharged in textile effluents when fixation to fibers is incomplete. Among these compounds, azo dyes are especially important because of their extensive industrial use, persistence in water, and potential ecotoxicological and health impacts associated with the parent molecules and their transformation products [1]. Naphthol Blue Black (NBB), an anionic diazo dye, belongs to this problematic group and represents a relevant model contaminant for evaluating advanced treatment strategies aimed at saline and chemically complex wastewaters [2], [3], [4].

Among advanced oxidation technologies, sonochemistry has attracted sustained interest because acoustic cavitation can generate highly reactive conditions in water without the need for direct light irradiation and, in its simplest form, without added catalysts [5], [6]. The collapse of cavitation bubbles produces localized extreme temperatures and pressures that promote water sonolysis and the formation of reactive species, chiefly hydroxyl radicals, while the surrounding bubble–liquid interfacial region becomes the main reaction zone for many nonvolatile organic contaminants [7], [8]. For azo dyes in particular, high-frequency ultrasound is often more effective than low-frequency irradiation because chemical effects become more dominant, leading to faster color removal and oxidative transformation [7], [8], [9].

NBB has already proven to be a useful probe molecule for dissecting the behavior of high-frequency sonochemical systems. Earlier studies showed that its degradation is strongly dependent on acoustic frequency, power density, and initial concentration, and that efficient conversion can be achieved at frequencies in the several-hundred-kilohertz range [2], [3]. Additional work demonstrated that sonochemical performance toward NBB can be intensified by coupling ultrasound with persulfate, confirming the importance of reactive-radical chemistry in controlling the degradation pathway [4]. These findings establish NBB as an appropriate substrate for investigating how changes in water chemistry alter high-frequency sonolysis.

A critical unresolved issue, however, is that most sonochemical studies are still interpreted from experiments performed in ultrapure or weakly mineralized water, whereas real waters and industrial effluents contain appreciable amounts of inorganic ions. This matters because salts are now known to influence sonolysis through several coupled mechanisms, including changes in ionic strength, contaminant partitioning toward the bubble interface, radical interception, dissolved gas behavior, and the number and activity of cavitation bubbles [10], [11]. Recent synthesis of the field has emphasized that salt effects are highly contaminant-specific and cannot be reduced to a simple *“salting-out”* explanation; depending on the ion and operating window, the same electrolyte may accelerate, suppress, or even reverse degradation trends [10]. In parallel, a dedicated review of sonochemistry in urine, mineral water, and seawater concluded that ion-rich matrices can either impair or enhance pollutant removal, with the outcome depending strongly on pollutant structure, concentration, and matrix composition [12]. Recent studies also confirm that ionic composition can substantially affect ultrasonic treatment in synthetic environmental matrices [13].

For NBB, this matrix problem is especially relevant because prior studies already hinted that salts and complex waters can profoundly modify degradation kinetics. Although hydroxyl radical production generally decreases markedly when the ultrasonic frequency is increased beyond its optimum intermediate-frequency range [7], this decrease does not imply the disappearance of chemically active cavitation at megahertz frequencies. Previous experimental studies from our group demonstrated sonochemical activity at 1700 kHz through KI and Fricke dosimetries, H_2_O_2_ formation, cavitation-field characterization, and pollutant degradation [15], [16]. In addition, NBB sonolysis at 1700 kHz was shown to remain sensitive to salts, complex water matrices, and persulfate [14]. These studies demonstrate that chemically active cavitation persists at 1700 kHz. Yet a systematic comparison of the major simple electrolytes most relevant to saline waters, i.e., chloride, bicarbonate, nitrate, and sulfate, under a single high-frequency operating condition, and in direct relation to real mineral water and seawater, is still lacking. As a result, the mechanistic hierarchy of salt effects on NBB remains insufficiently resolved, particularly in terms of how ion identity interacts with pollutant loading and how single-salt behavior translates into authentic multicomponent matrices [3], [10], [12], [17]. Unlike our previous study at 1700 kHz [14], the present work systematically resolves the concentration-dependent effects of four inorganic salts at 585 kHz and two NBB loadings, and directly relates the single-salt responses to those observed in natural mineral water and seawater.

Accordingly, the aim of the present study was to investigate the sonochemical degradation of NBB at 585 kHz in the presence of representative inorganic salts and natural water matrices and to interpret the observed ion-specific effects. The roles of NaCl, KNO_3_, Na_2_SO_4_, and NaHCO_3_ were examined over a wide concentration range at two initial NBB concentrations, and the resulting trends were compared with degradation behavior in a bicarbonate-rich Ca-Mg mineral water and seawater. By combining single-salt and real-matrix experiments under identical acoustic conditions, this work seeks to identify which ionic environments promote NBB oxidation, which induce inhibition or dual behavior, and why realistic saline backgrounds may depart from the behavior predicted from isolated electrolytes alone.

## 2 Materials and methods

### 2.1 Chemicals and water matrices

Naphthol Blue Black (NBB, C_22_H_14_N_6_Na_2_O_9_S_2_, molar mass: 616.49 g/mol) was selected as the model pollutant. NaCl, Na_2_SO_4_, NaHCO_3_, and KNO_3_ (Sigma-Aldrich) were used as representative inorganic salts. All stock solutions and single-salt experimental solutions were prepared with ultrapure water.

For the real matrix experiments, a natural mineral water and a seawater sample were used. The mineral water had a pH of 6.9 and contained: Ca^2+^ = 59 mg/L, Mg^2+^ = 45 mg/L, Na^+^ = 15 mg/L, K^+^ = 2 mg/L, Cl^−^ = 22 mg/L, SO_4_^2−^ = 40 mg/L, HCO_3_^−^ = 378.2 mg/L, and no detectable nitrate and nitrite. Seawater was collected from the Mediterranean coast of Annaba, Algeria, and used as the natural saline matrix. The seawater exhibited the following characteristics: pH 8.1, Ca^2+^ = 0.4 g/L, Mg^2+^ = 1.3 g/L, Na^+^ = 11.0 g/L, Cl^−^ = 20.0 g/L, and SO_4_^2−^ = 3.0 g/L.

### 2.2 Sonochemical experiments

Sonochemical runs were performed in a jacketed cylindrical glass cell with a total volume of 500 mL. Temperature was monitored continuously by a thermocouple placed directly in the liquid phase and controlled at 25 ± 1 °C by circulation of a cooling fluid through the reactor jacket. Ultrasonication at 585 kHz was applied from the bottom of the reactor using a Meinhardt multifrequency transducer (model E/805/T/M) with an active emitting surface of 5.3 cm in diameter. For the degradation experiments reported here, a working volume of 300 mL was employed. The acoustic power dissipated in the liquid phase was determined by the conventional calorimetric method [18], [19], [20], [21]. Based on these measurements, the salt-effect experiments were carried out at an acoustic intensity of 3.58 W/cm^2^ under the selected 585 kHz operating condition.

Stock and working solutions of NBB were prepared at the desired initial concentrations and transferred to the reactor before ultrasonication. The initial NBB concentrations used in the single-salt experiments were 5 mg/L (8.11 × 10^−6^ M) and 30 mg/L (4.87 × 10^−5^ M). For the natural-water experiments, the investigated range of 5–80 mg/L corresponded to 8.11 × 10^−6^–1.30 × 10^−4^ M. NaCl was investigated over 0.2–1.0 M at initial NBB concentrations of 5 and 30 mg/L. Na_2_SO_4_ was investigated over 0.2–1.0 M at 5 mg/L NBB and 0.1–1.0 M at 30 mg/L NBB. Bicarbonate experiments were initiated at pH 8.3. For 5 mg/L NBB, two NaHCO_3_ concentration intervals were investigated, namely 0.01–0.15 M and 0.2–1.0 M, to examine the effect of bicarbonate over a broad concentration range with greater resolution at low concentrations. For 30 mg/L NBB, NaHCO_3_ concentration was varied from 0.1 to 1.0 M. Nitrate experiments were carried out with KNO_3_ over the range 0.1−1.0 M at 5 and 30 mg/L NBB. To assess the behavior of realistic saline environments, additional experiments were performed in mineral water and seawater with initial NBB concentrations ranging from 5 to 80 mg/L.

At selected time intervals, aliquots were withdrawn from the reaction medium, and the residual dye concentration was quantified by UV–visible spectrophotometry (Lightwave II) at 620 nm, corresponding to the dye’s maximum absorbance in the visible region.

Normalized NBB concentration was expressed as C/C_0_, where C_0_ is the initial NBB concentration and C is the concentration at sonication time t.

Initial degradation rates were calculated from the slope of the initial linear portion of the NBB concentration–time profiles.

All experiments were carried out in triplicate, and the reported values are presented as mean ± standard deviation where applicable. Differences among experimental conditions are interpreted descriptively in terms of their magnitude, reproducibility, and persistence over the sonication profile.

## 3 Results and discussion

### 3.1 Effect of NaCl

NaCl produced an overall concentration-dependent enhancement of NBB sonolysis (Fig. 1). At 5 mg/L, 0.2 M NaCl produced little change relative to the salt-free control during the early stage, whereas 0.5–1.0 M progressively accelerated NBB disappearance. A similar trend was observed at 30 mg/L, where the beneficial effect became more evident as NaCl concentration increased. The initial-rate data support this pattern: at 5 mg/L the rate increased from approximately 0.41 mg/L·min without NaCl to 0.52 mg/L·min at 1.0 M, while at 30 mg/L it increased from approximately 0.87 to 1.05 mg/L·min.

**Fig. 1:**
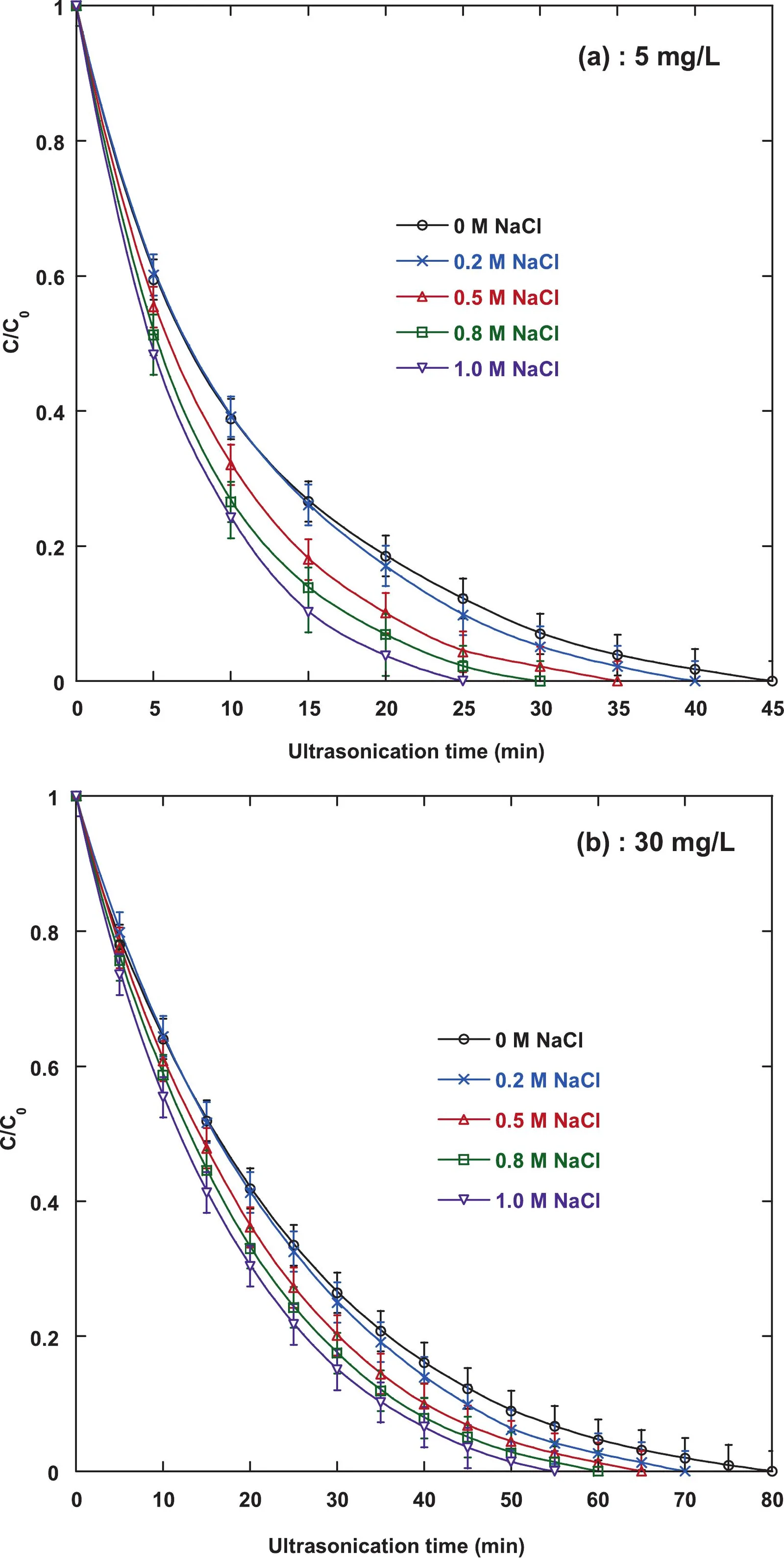

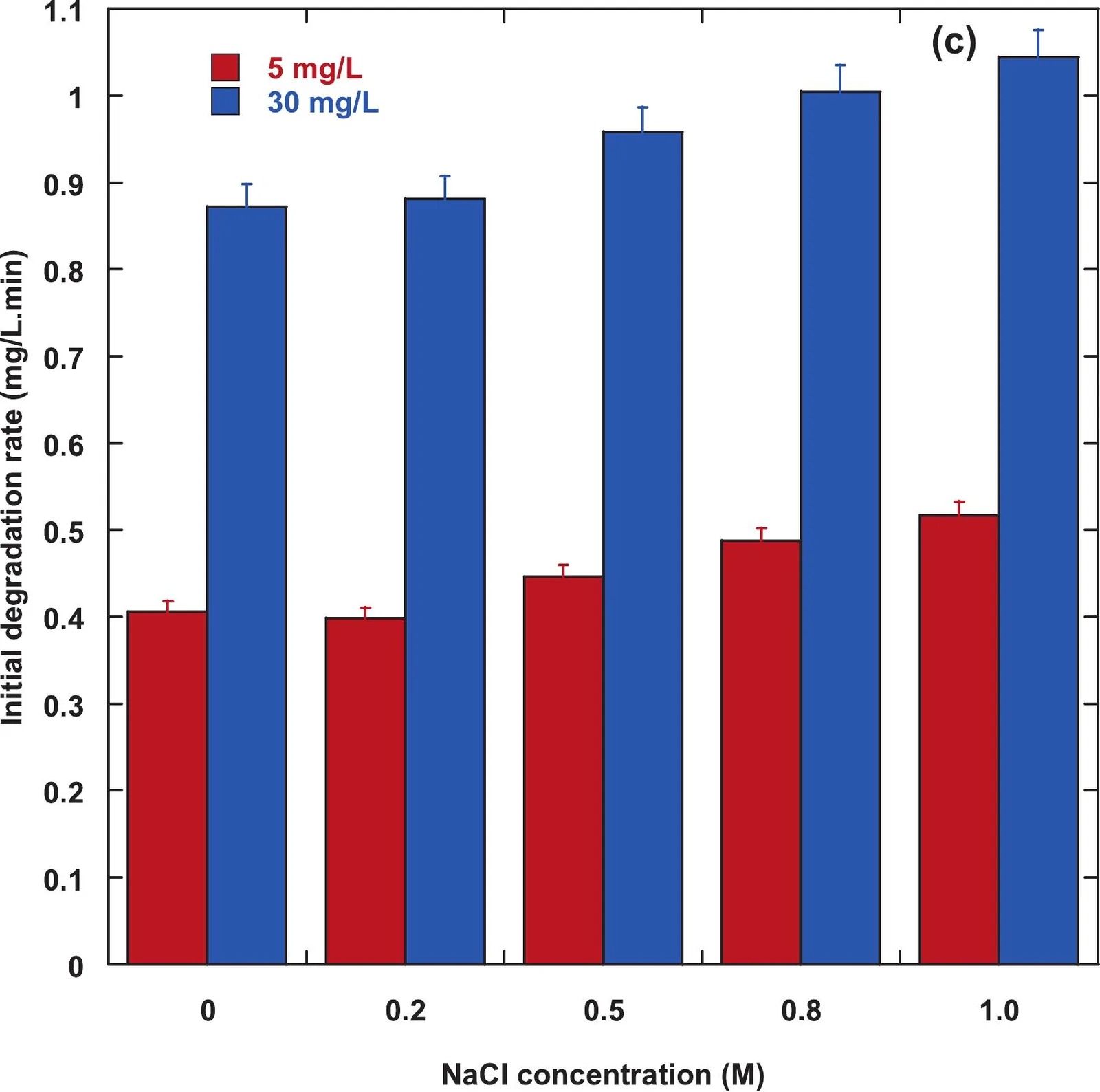
Effect of NaCl on the sonochemical degradation of NBB at 585 kHz and 3.58 W/cm^2^: **(a)** degradation profiles at 5 mg/L NBB, **(b)** degradation profiles at 30 mg/L NBB, and **(c)** evolution of the initial degradation rate as a function of NaCl concentration. The initial NBB concentrations of 5 and 30 mg/L correspond to 8.11 × 10^−6^ and 4.87 × 10^−5^ M, respectively.

Increasing NaCl concentration can modify solute partitioning toward the bubble–liquid interface, where nonvolatile compounds are exposed to high radical concentrations [10], [22]. Salts can also modify gas solubility, bubble population, collapse dynamics, and bulk sonochemical yields [22], [23], [24]. Changes in surface tension may further influence bubble growth and collapse [10], [22], [23]; however, surface tension was not measured here, so its contribution cannot be quantified independently.

Chloride-specific radical chemistry may also contribute. Under sonochemical conditions, Cl^−^ can participate in secondary chlorine-radical chemistry following interaction with HO^•^, producing reactive chlorine species such as Cl^•^ and Cl_2_^•−^ [10], [25]. Because these species were not measured in the present study, their contribution is considered a plausible complementary pathway rather than a demonstrated mechanism. Likewise, pH was not continuously monitored in the present experiments, although our previous NBB study under the same acoustic conditions showed acidification from approximately pH 6 to 2.8 during sonication [2], which could favor chloride-derived radical chemistry.

Overall, the observed NaCl response is consistent with combined physicochemical cavitation, interfacial, and possible chloride-derived radical effects. Within the investigated range, the net effect became increasingly favorable above approximately 0.5 M, although individual neighboring concentrations should not be regarded as statistically resolved solely from their error bars.

### 3.2 Effect of Na_2_SO_4_

Na_2_SO_4_ produced a concentration- and loading-dependent response (Fig. 2). At 5 mg/L NBB, sulfate generally promoted degradation over the investigated range, although the differences among neighboring concentrations were modest and several error bars overlap. At 30 mg/L, the response was much more pronounced: degradation improved progressively up to 0.8 M Na_2_SO_4_ but was strongly inhibited at 1.0 M. The initial-rate data show the same pattern, increasing from approximately 1.08 mg/L·min in the salt-free system to 1.46 mg/L·min at 0.8 M before decreasing to approximately 0.92 mg/L·min at 1.0 M.

**Fig. 2:**
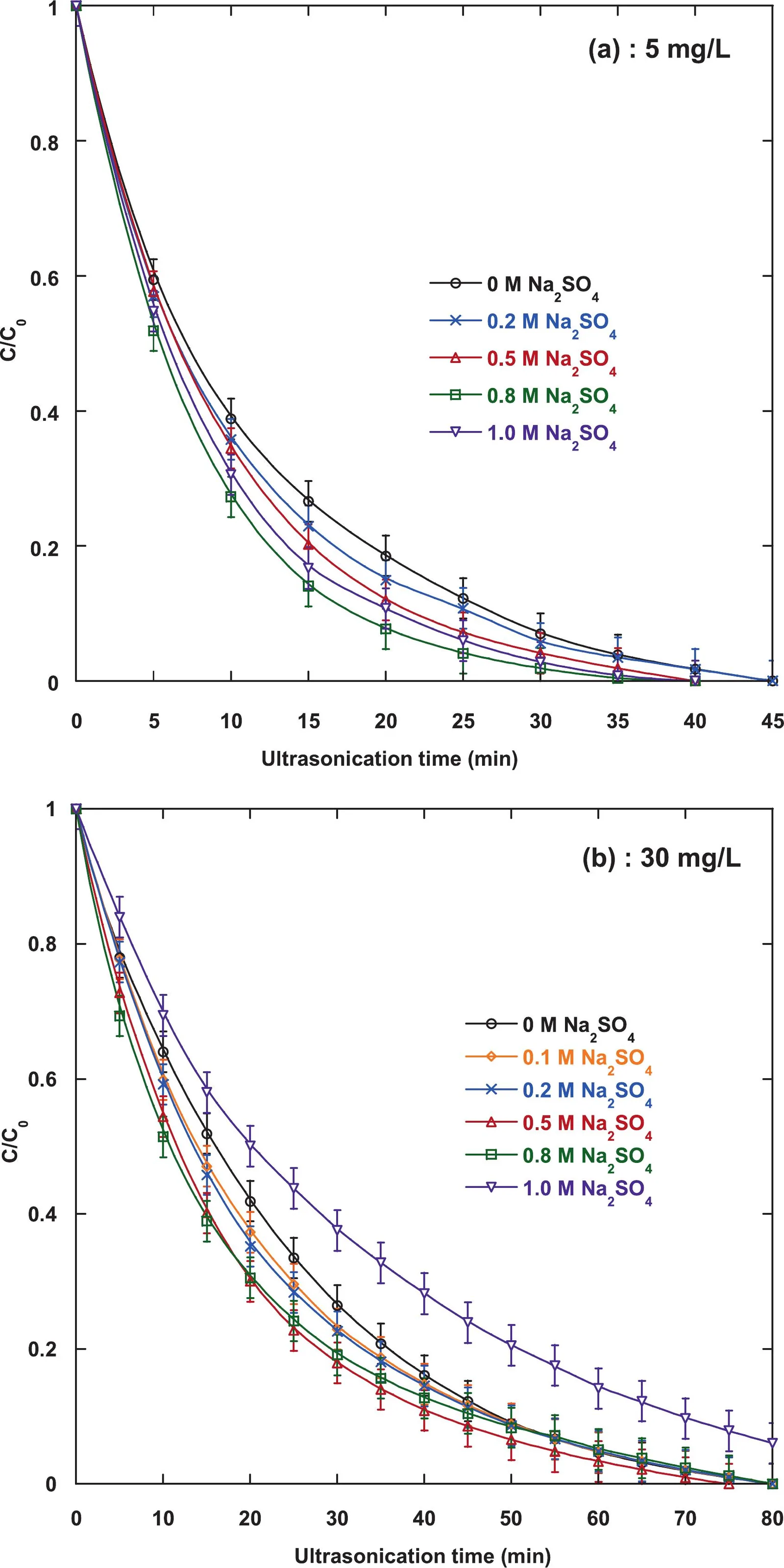

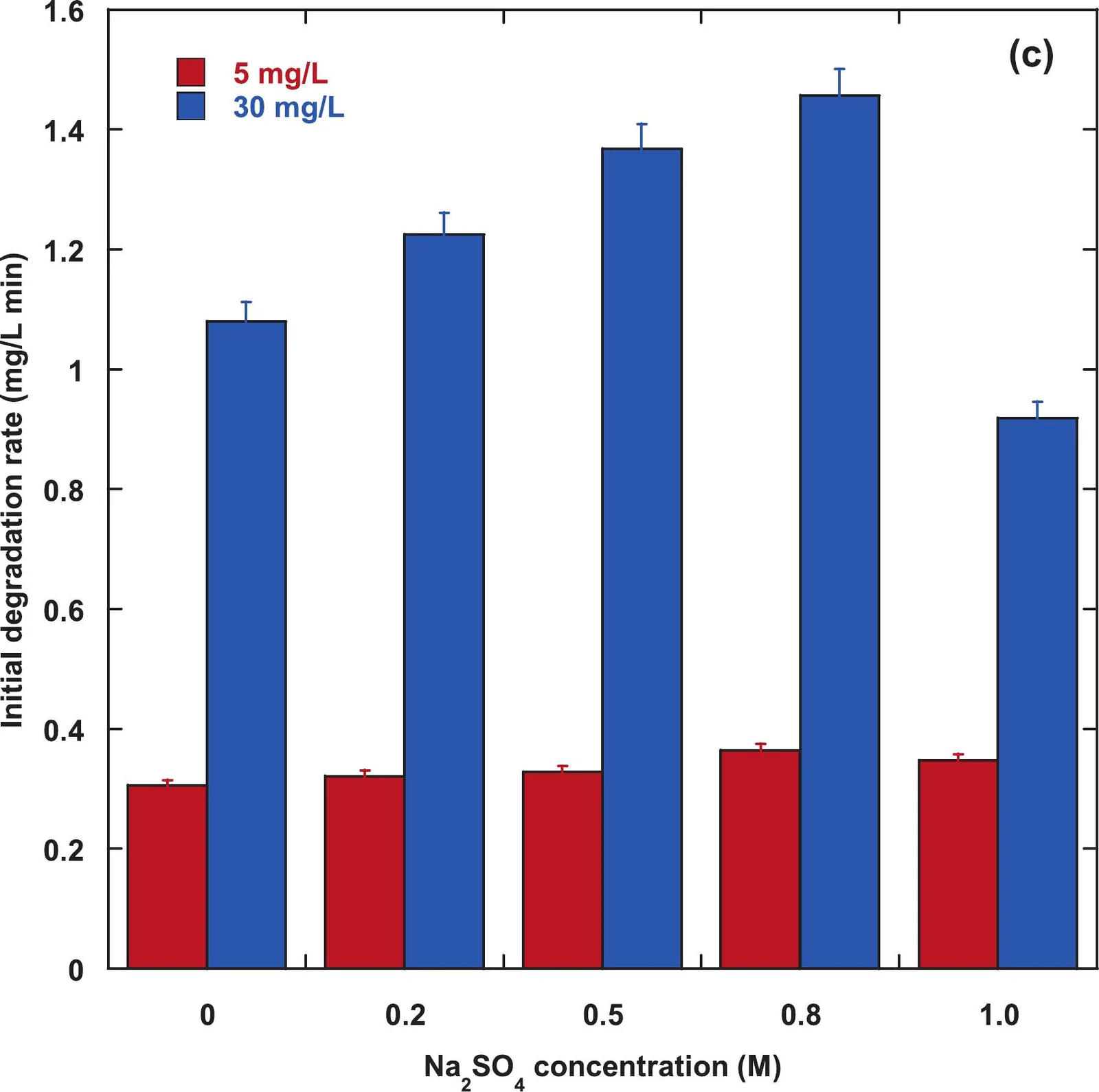
Effect of Na_2_SO_4_ on the sonochemical degradation of NBB at 585 kHz and 3.58 W/cm^2^: (a) degradation profiles at 5 mg/L NBB, (b) degradation profiles at 30 mg/L NBB, and (c) evolution of the initial degradation rate as a function of Na_2_SO_4_ concentration. The initial NBB concentrations of 5 and 30 mg/L correspond to 8.11 × 10^−6^ and 4.87 × 10^−5^ M, respectively.

Because no persulfate or other sulfate-radical precursor was introduced in these experiments, the Na_2_SO_4_ response is not attributed here to SO_4_^•−^ formation. The observed behavior is instead discussed in terms of literature-reported physicochemical effects of dissolved salts on interfacial accessibility and cavitation [10], [22], [23], [26]. Moderate sulfate concentrations may improve NBB accessibility to the interfacial reaction zone, whereas excessive electrolyte loading can alter gas behavior, bubble population, and the chemical productivity of cavitation collapse [10], [22], [23], [26].

On a concentration basis, 1.0 M Na_2_SO_4_ corresponds to a nominal ionic strength of 3.0 M, whereas 1.0 M NaCl corresponds to 1.0 M. At these high concentrations, however, substantial non-ideal solution behavior is expected; ionic strength is therefore used here only as a comparative descriptor. Because NaCl and Na_2_SO_4_ were not studied at matched ionic strengths, their different responses cannot be attributed solely to ionic strength. The high-concentration sulfate inhibition is therefore interpreted as the combined result of electrolyte loading, sulfate-specific interactions, cavitation modification, and pollutant accessibility.

Thus, Na_2_SO_4_ promoted NBB sonolysis over the investigated concentration range at 5 mg/L, whereas its effect at 30 mg/L shifted from enhancement up to 0.8 M to inhibition at 1.0 M.

### 3.3 Effect of NaHCO_3_

NaHCO_3_ produced the strongest concentration-loading dependence among the investigated salts (Fig. 3). At 5 mg/L NBB, low bicarbonate concentrations enhanced sonolysis, with the strongest response around 0.05 M and promotion remaining evident up to approximately 0.2 M. This behavior is inconsistent with bicarbonate acting only as an HO^•^ scavenger and is compatible with formation of carbonate radicals:

(1) $${\text{HCO}}_{\text{3}}^{-}+{\;\text{HO}}^{∙}\to {\;\text{CO}}_{\text{3}}^{∙-}+{\;\text{H}}_{2}\text{O}$$

**Fig. 3:**
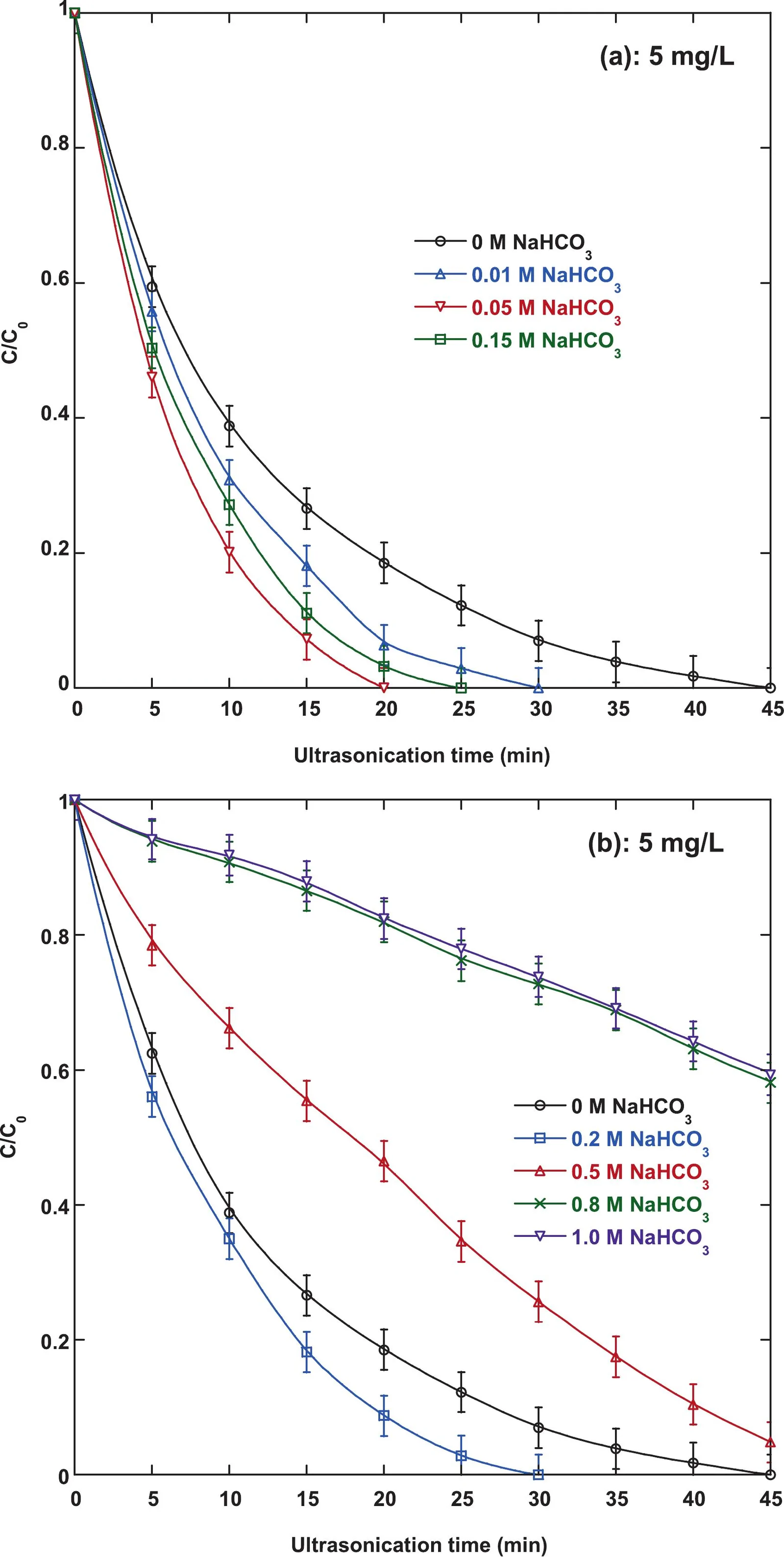

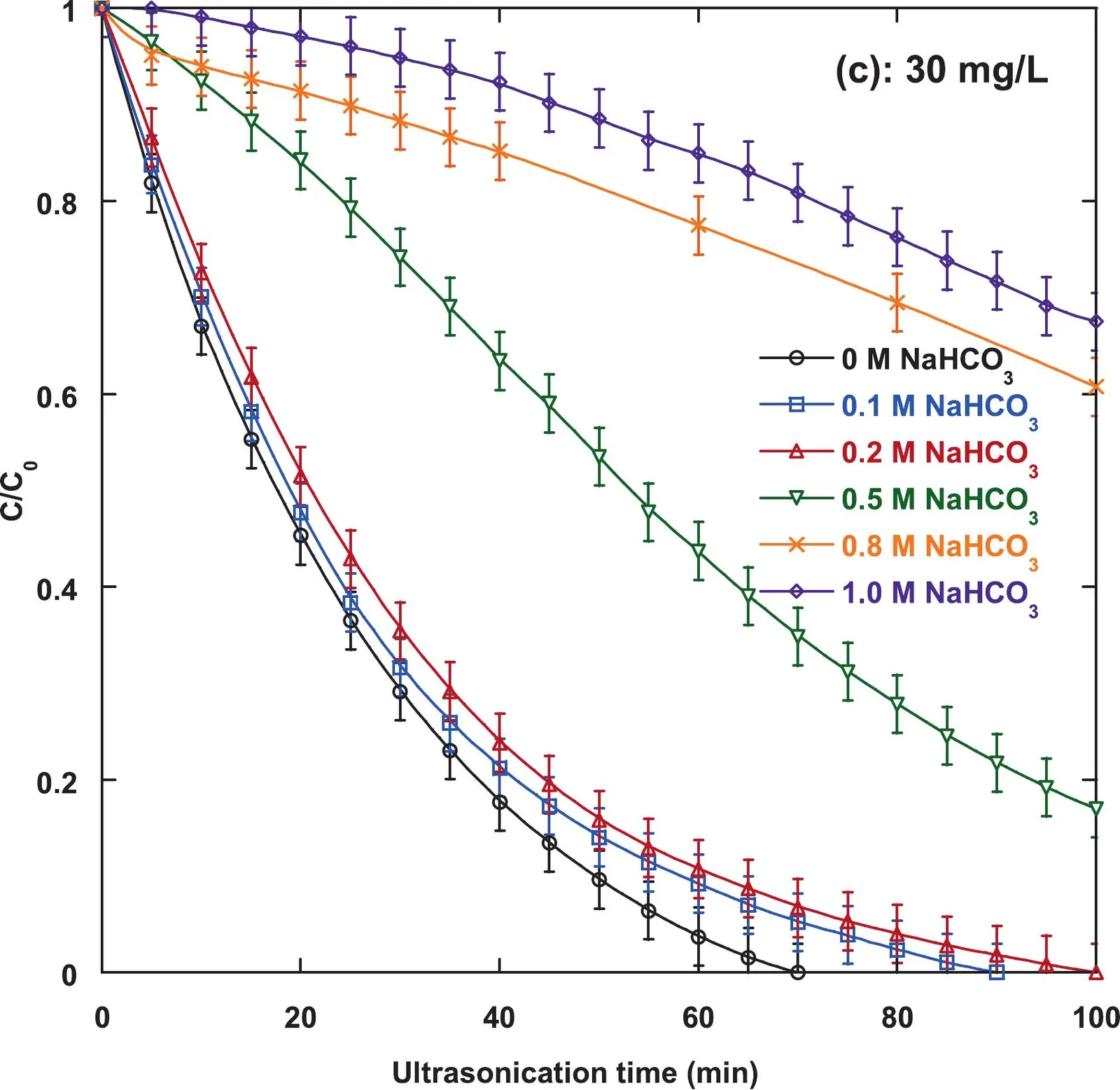
Effect of NaHCO_3_ on the sonochemical degradation of NBB at 585 kHz and 3.58 W/cm^2^: (a) degradation profiles at 5 mg/L NBB in the low NaHCO_3_ concentration range, (b) degradation profiles at 5 mg/L NBB in the high NaHCO_3_ concentration range, and (c) degradation profiles at 30 mg/L NBB at different NaHCO_3_ concentrations. The initial NBB concentrations of 5 and 30 mg/L correspond to 8.11 × 10^−6^ and 4.87 × 10^−5^ M, respectively.

CO_3_^•−^ is less reactive but longer lived and more selective than HO^•^, allowing oxidation farther from the immediate bubble–liquid interface [10], [27], [28], [29]. At low pollutant loading, partial conversion of HO^•^ into CO_3_^•−^ may therefore remain productive. Because CO_3_^•−^ was not directly measured, this pathway is invoked as a literature-supported interpretation rather than as direct mechanistic evidence.

Above approximately 0.2 M NaHCO_3_, the response reverses. At 0.5–1.0 M, degradation was increasingly inhibited, consistent with increasing HO^•^ scavenging and a greater relative contribution of the less reactive carbonate-radical pathway. Under the pH 8.3 conditions used here, bicarbonate may also influence pollutant speciation, interfacial interactions, and radical reactivity [17], [30].

At 30 mg/L NBB, all investigated bicarbonate concentrations were detrimental, with strong inhibition at 0.5–1.0 M. The higher pollutant loading increases demand for the primary oxidizing flux, making diversion of HO^•^ into less reactive secondary radicals less favorable. The data therefore support a concentration-loading crossover in which bicarbonate can assist dilute NBB sonolysis at low concentration but becomes inhibitory as either bicarbonate or pollutant loading increases.

### 3.4 Effect of KNO_3_

KNO_3_ produced a generally favorable but comparatively modest effect on NBB sonolysis (Fig. 4). At initial NBB concentrations of 5 mg/L (8.11 × 10^−6^ M) and 30 mg/L (4.87 × 10^−5^ M), KNO_3_ generally accelerated NBB degradation relative to the salt-free control. However, the differences among individual KNO_3_ concentrations were relatively small and several error bars overlap; therefore, the neighboring nitrate concentrations should not be interpreted as statistically distinct. The overall tendency was more evident at 30 mg/L, where the initial rate increased from approximately 0.80 mg/L·min at 0.1 M to 1.06 mg/L·min at 1.0 M.

**Fig. 4:**
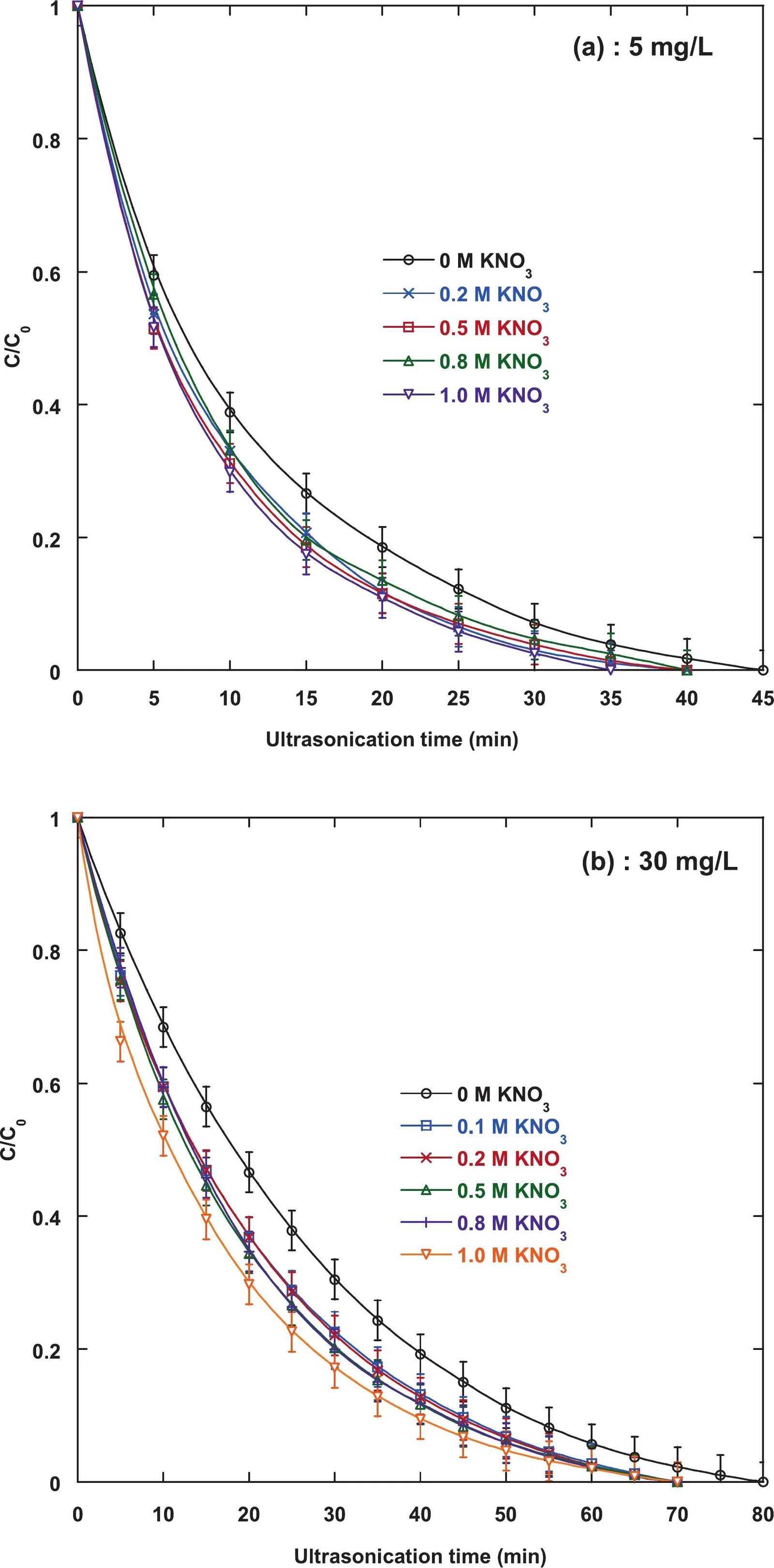

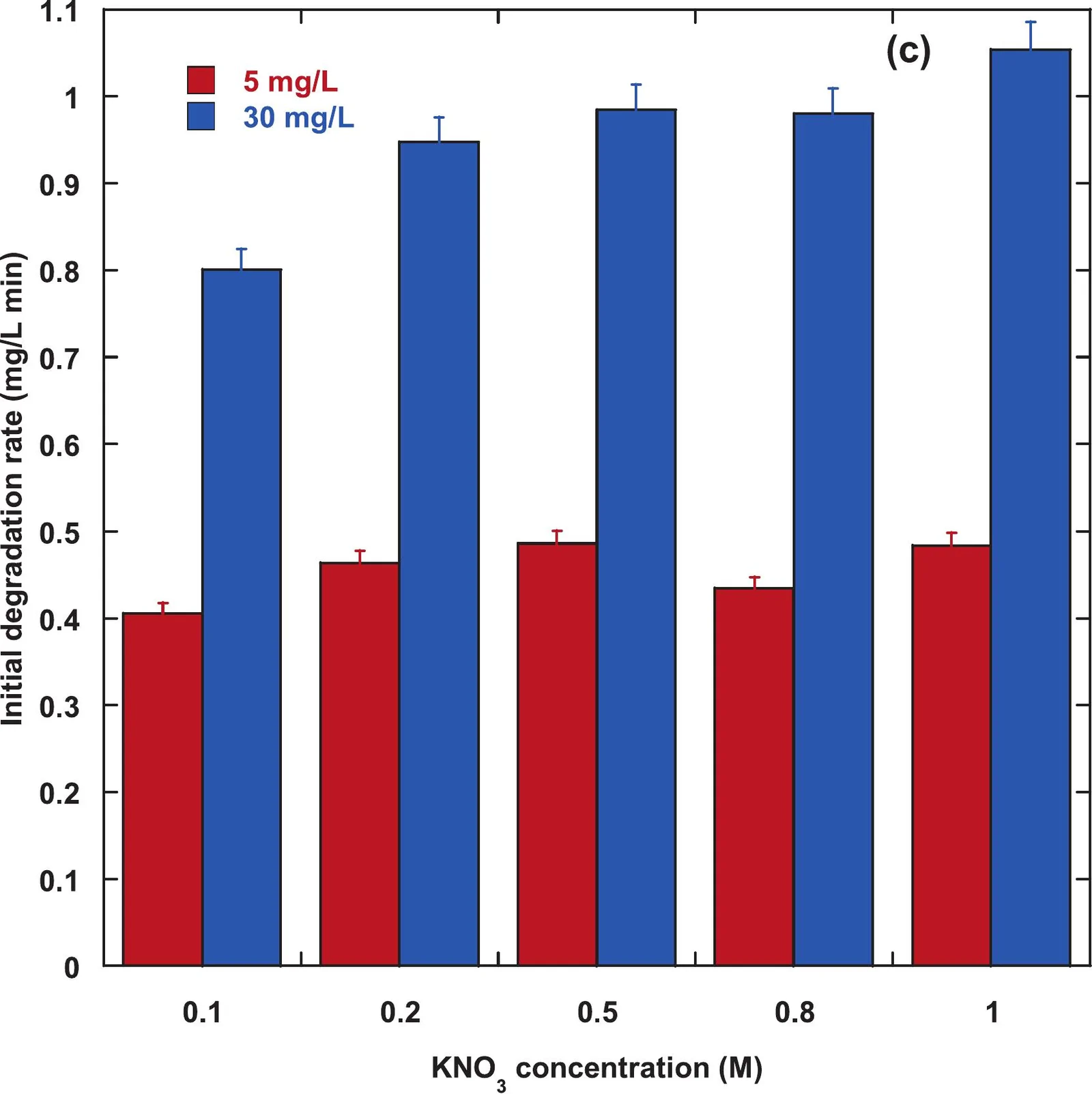
Effect of KNO_3_ on the sonochemical degradation of NBB at 585 kHz and 3.58 W/cm^2^: (a) degradation profiles at 5 mg/L NBB, (b) degradation profiles at 30 mg/L NBB, and (c) evolution of the initial degradation rate as a function of KNO_3_ concentration. The initial NBB concentrations of 5 and 30 mg/L correspond to 8.11 × 10^−6^ and 4.87 × 10^−5^ M, respectively.

Nitrate can interact with sonochemically generated HO^•^ [31], [32], and reactive nitrogen species may also form during sonolysis of aerated water [33], [34]. However, none of these species was measured in the present study. The degradation data therefore do not establish a nitrate-specific reaction pathway. The modest enhancement observed with KNO_3_ is more conservatively interpreted as an empirical matrix effect that may involve combined ionic-strength, interfacial, cavitation, and radical-interception phenomena. The nitrate response is also pollutant-specific, as different effects have been reported for other sonolyzed compounds [35]. Under the present conditions, nitrate produced a modest overall enhancement of NBB sonolysis without the strong inhibitory behavior observed for concentrated sulfate or bicarbonate.

### 3.5 Natural matrix enhancement in mineral water and seawater

Natural water composition had a favorable overall effect on NBB sonolysis (Fig. 5). At both 5 and 30 mg/L, the degradation profiles followed the general order seawater > mineral water > ultrapure water. Although individual time points may overlap within experimental variability, this ordering persists over substantial portions of the degradation profiles and is also reflected in the initial-rate data.

**Fig. 5:**
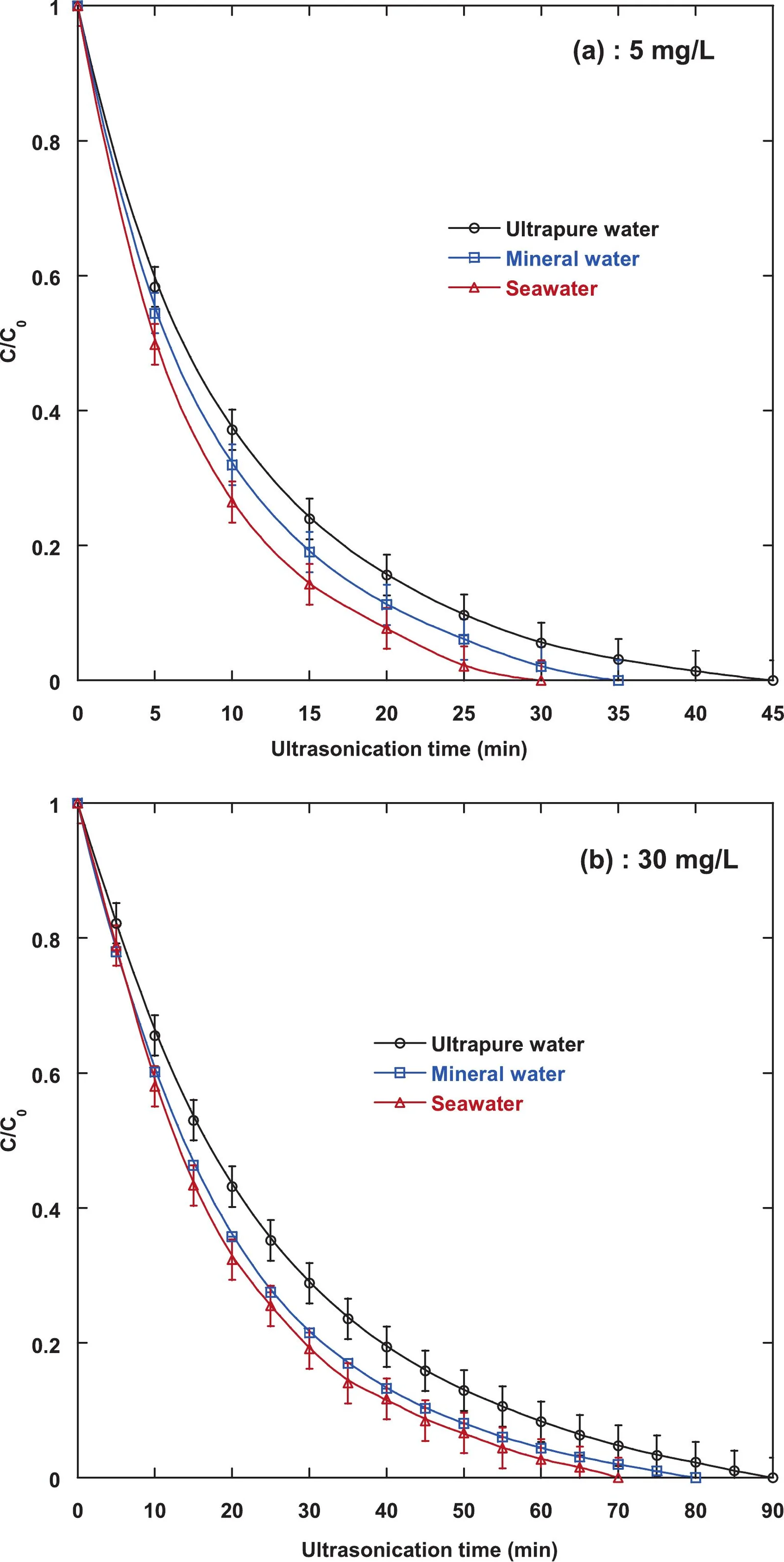

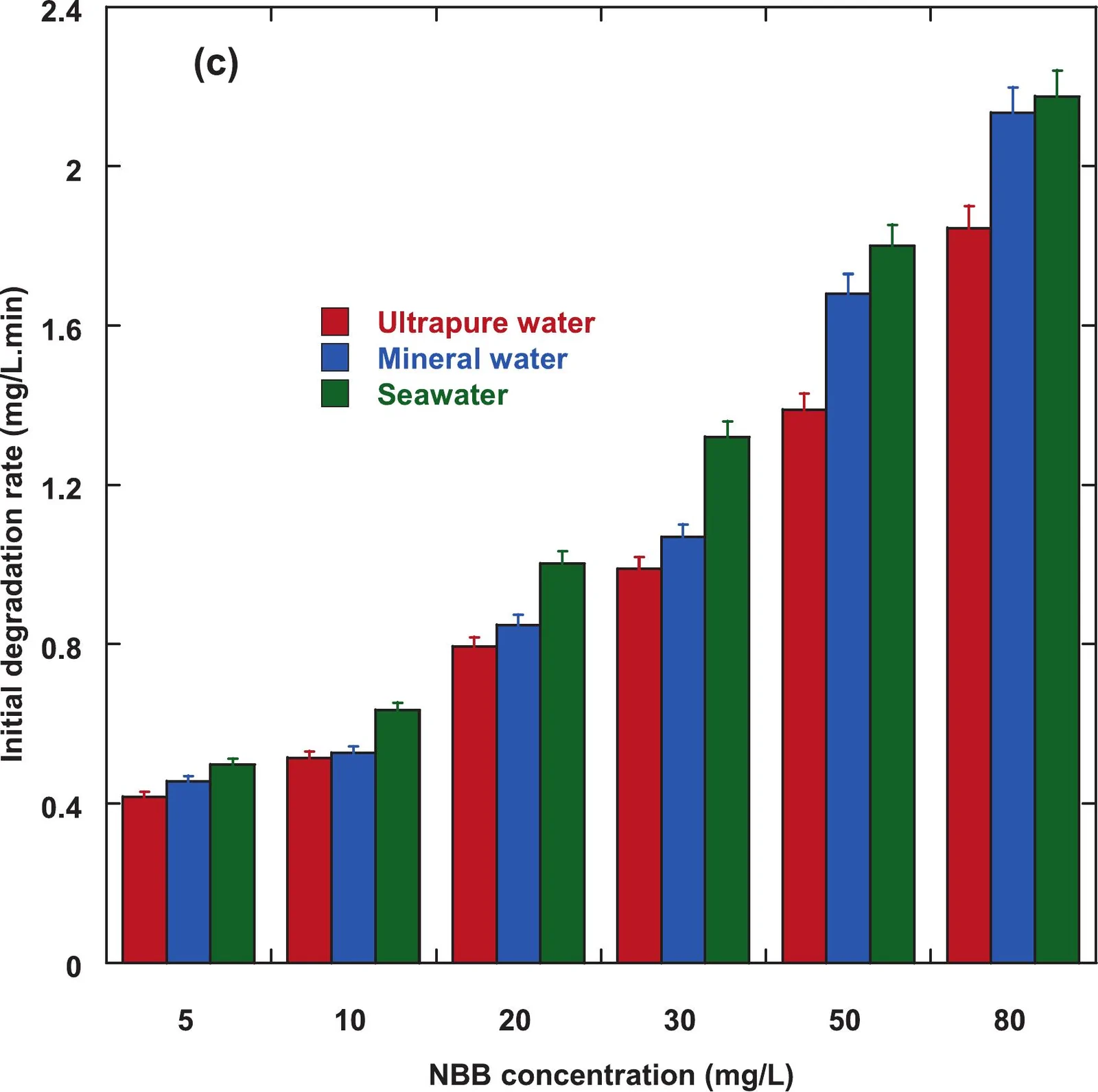
Effect of real water matrices on the sonochemical degradation of NBB at 585 kHz and 3.58 W/cm^2^: (a) degradation profiles in ultrapure water, mineral water, and seawater at 5 mg/L, (b) degradation profiles in ultrapure water, mineral water, and seawater at 30 mg/L, and (c) evolution of the initial degradation rate as a function of initial dye concentration in the different matrices. The initial NBB concentrations of 5, 10, 20, 30, 50, and 80 mg/L correspond to 8.11 × 10^−6^, 1.62 × 10^−5^, 3.24 × 10^−5^, 4.87 × 10^−5^, 8.11 × 10^−5^, and 1.30 × 10^−4^ M, respectively.

The mineral water was dominated by bicarbonate (∼6.2 mM), with much lower concentrations of Cl^−^ (∼0.62 mM) and SO_4_^2−^ (∼0.42 mM) and millimolar Ca^2+^ and Mg^2+^. Its bicarbonate concentration is therefore far below the 0.5–1.0 M range that strongly inhibited NBB degradation in the single-salt experiments. The beneficial response of this matrix is consequently consistent with a dilute multicomponent system rather than with concentrated NaHCO_3_ behavior [12], [17], [28], [29], [36].

Seawater produced the strongest enhancement and represents a high-ionic-strength, chloride-rich multicomponent matrix. Its behavior is qualitatively consistent with the favorable chloride response observed in the single-salt experiments, although seawater cannot be treated as concentrated NaCl because sulfate, Mg^2+^, Ca^2+^, and the remaining components of the natural matrix contribute simultaneously [12], [37].

The persistence of the real-matrix advantage at 30 mg/L indicates that the effect is not confined to dilute NBB solutions. These results therefore show that authentic mineral and saline matrices can remain compatible with, and in the present case enhance, high-frequency NBB sonolysis. Importantly, the response of each natural water reflects its complete ionic composition rather than the action of a single constituent.

### 3.6 Integrative interpretation of matrix effects

Taken together, the observed trends are consistent with three literature-supported contributions: (i) changes in NBB accessibility to the bubble–liquid interfacial reaction zone, (ii) ion-dependent changes in radical chemistry, and (iii) modification of the cavitation environment [2], [10], [12], [14], [22], [28], [36], [37]. Their individual contributions cannot be separated quantitatively from the present degradation data and likely depend on ion identity, electrolyte concentration, and pollutant loading.

High ionic strength may influence solvation and interfacial partitioning of organic solutes [10], while dissolved salts can also alter gas solubility and bubble dynamics [22], [23], [26]. These quantities were not measured in the present work; therefore, no specific contribution from NBB aggregation, ion pairing, or complex formation is inferred.

Experimentally, chloride showed the most persistent promotion, nitrate a weaker positive trend, sulfate shifted from promotion to inhibition at high salt concentration, and bicarbonate displayed the strongest concentration-loading crossover. Because NaCl and Na_2_SO_4_ were not tested at matched ionic strengths, the respective contributions of ion identity and ionic strength cannot be separated. The natural-water results likewise represent the combined effect of the complete ionic matrix rather than any single constituent.

### 3.7 Implications for treatment design and environmental relevance

The present results show that ultrapure water alone may not be a sufficient surrogate for predicting NBB sonolysis in mineralized or saline matrices. Under the present laboratory conditions, both mineral water and seawater performed better than ultrapure water, showing that salinity and mineralization did not impair high-frequency sonochemical treatment of NBB [12], [36], [37]. Water chemistry should therefore be considered explicitly when defining operating conditions.

These favorable matrix effects should not, however, be interpreted as evidence that high-frequency sonochemistry is already technologically mature for large-scale treatment. Important challenges remain in the design of energy-efficient continuous-flow reactors, maintenance of suitable acoustic-field distributions during scale-up, and efficient transfer of acoustic energy to the liquid phase [5], [20]. The present results address a complementary issue by demonstrating that high-frequency sonolysis can remain effective in authentic saline and mineralized waters rather than only in idealized ultrapure-water systems.

Pollutant loading must also be considered together with matrix composition because normalized NBB removal became slower as the initial concentration increased. Consequently, required treatment time and operating conditions should not be extrapolated from dilute pure-water experiments without matrix-specific verification. The present results support matrix-aware evaluation of sonochemical treatment while recognizing the remaining reactor-scale and energy-efficiency limitations.

## 4 Conclusions

The sonochemical behavior of NBB at 585 kHz was strongly dependent on ion identity, salt concentration, and pollutant loading. NaCl produced the most persistent enhancement, KNO_3_ showed a weaker positive effect, Na_2_SO_4_ exhibited a loading-dependent response, with the strongest enhancement around 0.8 M and inhibition relative to the salt-free control at 1.0 M for 30 mg/L NBB, and NaHCO_3_ shifted from promotion at low concentration and low NBB loading to strong inhibition under more demanding conditions. These experimental responses are consistent with, but do not independently demonstrate, coupled contributions from interfacial pollutant accessibility, radical chemistry, and cavitation behavior.

Importantly, both natural matrices enhanced NBB sonolysis relative to ultrapure water, with seawater giving the strongest overall response. This demonstrates that realistic saline and mineralized waters can support NBB sonolysis under the present high-frequency conditions and that results obtained exclusively in ultrapure water may not predict behavior in authentic matrices. The results therefore provide an experimental basis for matrix-aware evaluation of sonochemical treatment while recognizing that mechanistic confirmation and practical implementation will require additional radical-specific measurements and advances in reactor scale-up and energy efficiency.

## CRediT authorship contribution statement

**Hamza Ferkous:** Investigation, Data curation, Validation, Visualization. **Muthupandian Ashokkumar:** Conceptualization, Validation, Supervision, Visualization. **Oualid Hamdaoui:** Investigation, Conceptualization, Data curation, Methodology, Formal analysis, Resources, Validation, Project administration, Supervision, Funding acquisition, Visualization, Writing – original draft, Writing – review & editing.

## Declaration of competing interest

The authors declare that they have no known competing financial interests or personal relationships that could have appeared to influence the work reported in this paper.
